# Bibliometric Study of the National Scientific Production of All Peruvian Schools of Dentistry in Scopus

**DOI:** 10.1155/2021/5510209

**Published:** 2021-04-16

**Authors:** Frank Mayta-Tovalino, Josmel Pacheco-Mendoza, Ana Diaz-Soriano, Fernando Perez-Vargas, Arnaldo Munive-Degregori, Silvia Luza

**Affiliations:** ^1^Postgraduate Department, CHANGE Research Working Group, Faculty of Health Sciences, Universidad Científica del Sur, Lima, Peru; ^2^Unidad de Investigación en Bibliometría, Universidad San Ignacio de Loyola, Lima, Peru; ^3^Academic Department, Faculty of Dentistry, Universidad Nacional Mayor de San Marcos, Lima, Peru; ^4^Postgraduate Department, Faculty of Letters and Human Sciences, Universidad Nacional Mayor de San Marcos, Lima, Peru

## Abstract

**Objective:**

To perform a bibliometric analysis of the national scholarly output of all dental schools in Peru in Scopus through a retrospective study after the promulgation of the Peruvian University Law 30220 in 2014.

**Methods:**

This was a descriptive, comparative, retrospective, and cross-sectional study. A search of the Scopus database was performed to identify scholarly output in dentistry between 2014 and 2019. A total of 287 scientific articles with affiliation with the dental faculties of Peruvian public and private universities were evaluated. The data was extracted from Scopus using a complex formula developed from the words of the thesaurus MeSh (Medline) and Emtree (base) with words related to dentistry combined with the AF-IDs of the Peruvian universities.

**Results:**

The Top 10 in the scholarly output of all the Peruvian public and private dental schools were as follows: firstly, the Universidad Peruana Cayetano Heredia (UPCH), with 79 scholarly outputs publications and 5.2 citations per article, followed by the Universidad Nacional Mayor de San Marcos (UNMSM) with 53 scholarly outputs publications and 2.6 citations per article, and in third place, the Universidad Cientifica del Sur (UCSUR) with 49 scholarly outputs publications. In general, it was found that, of the 30 Faculties of Dentistry in Peru, only 10 in the public and private universities have a scholarly output greater than 5 to belong to the top 10. In addition, it was found that, according to the share of publications per journal quartile by the CiteScore Percentile of all the public and private Peruvian Faculties of Dentistry, in 2019, it presented the highest number of scientific publications in all quartiles with 20,33,14 and 43 articles in the quartiles Q1, Q2, Q3, and Q4, respectively. The highest number of scientific publications was produced in 2019 with 20, 33, 14, and 43 articles in quartiles Q1, Q2, Q3, and Q4, respectively.

**Conclusion:**

The UPCH, UNMSM, and UCSUR dental schools were the most productive. Both public and private universities presented an evident increase in their scientific publications in Scopus after the promulgation of the University Law 30220 in 2014.

## 1. Introduction

Traditionally, bibliometrics is known as “the measurement of patterns in written communication” [1]. However, it involves not only the characterization of publications and citations but also the evaluation of variables that include the number of scientific publications and citations that each author, research group, or institution has over time [2]. For example, in the health sciences and especially in dentistry, bibliometric methods are used with the intention of quantifying scientific production [3]. These publications become indicators of important areas in research and, therefore, can also show trends in thematic areas by individual authors, research groups, universities, scientific societies, and countries [4].

Bibliometric studies are important at an institutional level because they allow identifying and evaluating scientific performance in order to provide accurate and objective information through a critical analysis of what has been published. Strategies can be established to improve and plan programs that develop and motivate research, allocate funding, and optimize resources for the benefit of the scientific community [1, 2, 5].

The creation of governmental institutions that promote and supervise the development of research in universities is the true engine of great changes, especially in countries undergoing both national and international scientific development. Universities are the main axis and have a great responsibility in innovating and adapting the promotion of researchers in order to continue with the production of knowledge, which is the main objective of a university [6]. On the other hand, scientometry, the science of evaluating scientific production in order to measure its progress, allows characterizing information to plan and promote the development of programs and optimize human resources (mentoring) [7–12]. In this sense, it is essential to determine the total production that a country has in relation to the world context. However, in some areas of health, there is little literature that allows critical analysis of the Latin American dental scientific literature in general.

Several years ago in 2014, in Peru, the new University Law No. 30220 was promulgated. At this time, the creation of the Superintendencia Nacional de Educación Superior Universitaria (SUNEDU) was formalized. This institution was created by the Peruvian State to evaluate and ensure the basic conditions of educational quality, with one of its main functions being the promotion of science and research through the scientific publications of Peruvian universities. SUNEDU has become the regulatory body to oversee that the resources of the public treasury are entirely devoted to improving the quality of education and research in Peru. Through the promulgation of University Law No. 30220, this institution seeks continuous improvement of the Peruvian university educational model, in both public and private universities, as well as the promotion of research teachers based on their academic-scientific production [13, 14].

Therefore, the aim of this study was to determine a bibliometric study of the national scholarly output of all dental schools in Peru in Scopus through a retrospective study after the promulgation of the Peruvian University Law 30220.

## 2. Methods

### 2.1. Study Design

**Figure 1:**
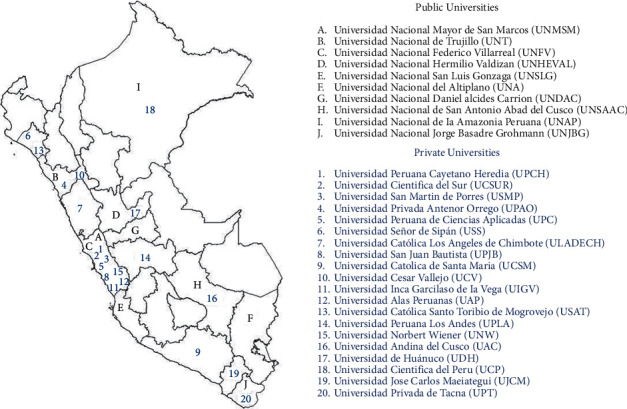
Geographical distribution of all the Faculties of Dentistry of the public and private universities of Peru.

A retrospective, cross-sectional, and descriptive study was carried out. All the dental faculties of Peru were evaluated (*N* = 30) of which 10 were national universities and 20 private universities distributed in the 25 departments of the Peruvian State (Figure 1).

### 2.2. Search Strategy

A search strategy was designed for all Peruvian universities that have dental schools and their respective scientific articles published between 2014 and 2019. The scientific production of each of the dental schools in Peru was extracted. For this, the information provided by the Scopus database corresponding to the profile of each university evaluated was used, and the institutional file of each university was used as a data source. The advanced search in Scopus involved the selection of each university file (AF-ID) combined with the SUBJTERMS codes of the All-Science Journal Classification (ASJC) on the Scopus source list in addition to the keywords extracted from the thesaurus MeSH (PubMed) and Emtree (Embase) (Appendix S1, S2).

The result of the complete search was exported to the SciVal bibliometric tool able to process the data and extract the results for each of the dimensions. SciVal is an online bibliometric analysis tool from Elsevier. It is a web-based analytical tool with unmatched power and flexibility that provides comprehensive access to the research performance of more than 14000 research institutions and their associated researchers from 230 nations worldwide. SciVal enables the visualization of research performance, comparison among peers, the development of strategic partnerships, the identification and analysis of emerging new research trends, and the creation of unique custom reports. Through a simple count, the scientific articles previously identified in the bibliometric analysis were assigned to each university, in which each institution was analyzed according to its identification code and abbreviation in Scopus (Appendix S3, S4).

### 2.3. University Selection

Peruvian universities were evaluated according to the institutional licensing process proposed by SUNEDU. The dental schools were distributed throughout the Peruvian territory and were classified as public and private (Figure 1). The analysis included the period from 2014 to 2019.

  Inclusion criteria:
(i) Public Peruvian Universities with Faculties of Dentistry
(ii) Private Peruvian Universities with Faculties of Dentistry
(iii) Public and private universities with and without institutional licensing by SUNEDU
  Exclusion criteria:
(i) Universities that do not have Scopus registration

### 2.4. Statistical Analysis

The data collected from all the dental schools were recorded in a Microsoft Excel spreadsheet for analysis. A descriptive evaluation of the numerical variables was used to obtain the means and standard deviations outlined by means of graphs bars. Additionally, the Scopus database (Scival) was used to perform this bibliometric analysis.

## 3. Results

### 3.1. Geographical Distribution of All the Faculties of Dentistry

It was found that Peru has 10 (33.4%) public universities with Faculties of Dentistry and 20 (66.6%) private universities with dentistry faculties distributed throughout the national territory and the Department of Lima (33.3%) has the highest concentration of dental schools in both public and private universities (Figure 1).

### 3.2. Scholarly Output in Public and Private Faculties of Dentistry by the Amount of International, National, and Institutional Collaboration (2014–2019)

The scholarly output of the public and private Faculties of Dentistry showed international collaboration of 54.9%, with a citation rate per publication of 26.8, while national collaboration was 17.8%. Finally, single authorship (no collaboration) represented only 7.7% of all the scholarly output in Scopus during 2014–2019 (Table 1).

### 3.3. Scholarly Output by Scopus Source of All Public and Private Peruvian Faculties of Dentistry

In relation to the scholarly output according to Scopus Source 2014–2019 of all public and private Peruvian Faculties of Dentistry, the Journal of Oral Research had the highest output with 41 scientific publications; however, it only had a quartile of Q4. On the other hand, the International Journal of Dentistry only had 9 scientific publications but was the journal with the highest quartile (Q2) of all the journals in which the Peruvian researchers usually publish (Table 2).

### 3.4. Share of Publications per Journal Quartile by CiteScore Percentile of All Public and Private Peruvian Faculties of Dentistry (2014–2019)

According to the share of publications per journal quartile by CiteScore Percentile of all public and private Peruvian Faculties of Dentistry, in 2019, the highest number of scientific publications in all quartiles was produced with 20, 33, 14, and 43 articles in the Q1 (top 25%), Q2 (top 26–50%), Q3 (top 51–75%), and Q4 quartiles (top 76–100%), respectively. Scientific production in the area of dentistry was found to represent only 2% (337 publications) of national production (17116 publications) during 2014–2019 (Table 3). However, if we analyze only the universities with a dental faculty (30 public and/or private universities in Peru), the area of dentistry represents 718 (4%), 944 (3%), 1,150 (3%), 1,226 (5%), 1,468 (5%), and 1,810 (6%) publications for the same 2014–2019 period, respectively.

### 3.5. Top 10 in Scholarly Output in Scopus of All the Peruvian Public and Private Dental Schools (2014–2019)

The top 10 in the scholarly output of all the Peruvian public and private dental schools were as follows: firstly, the Universidad Peruana Cayetano Heredia (UPCH) with 79 scholarly outputs publications with 5.2 citations per article, followed by the Universidad Nacional Mayor de San Marcos (UNMSM) with 53 scholarly outputs publications and 2.6 citations per article. In general, it was observed that of the 30 Faculties of Dentistry in Peru, only 10 in public and private universities have a scholarly output greater than 5 to belong to the top 10 (Table 4).

### 3.6. Top 3 Authors of Scholarly Output in Scopus of All Peruvian Public and Private Dental Schools (2014–2019)

On the other hand, it was possible to identify the Peruvian dentists with the highest scholarly output according to the Scopus data (2014–2019). The top 3 authors were Arriola-Guillén Luis Ernesto, Mayta-Tovalino Frank, and Mendoza-Azpur Gerardo, who are professors from private and national universities (Universidad Científica del Sur UCSUR and UNMSM), with 20, 14, and 14 scientific publications, respectively (Table 5).

### 3.7. Scholarly Output and International Collaboration (%) according to Publications in Top 25% Journal Percentiles by CiteScore Percentile (%) = Q1 Output in Top 25% Citation Percentiles (%) (2014–2019)

In summary, the UPCH, UNMSM, and UCSUR were found to have 51.9%, 15.4%, and 26.5% of publications in the top 25% journal percentiles by CiteScore, respectively, with these institutions being the most academically productive (Table 6).

Finally, Appendix S4 shows that the Peruvian Universities that most influence scientific production in Peruvian dentistry are the UPCH, UNMSM, and UCSUR, and these institutions have maintained sustained growth of scholarly output per year, 2019 being one of the most fruitful years with 24% (490), 23% (458), and 7% (134) of publications in dentistry. However, regarding the total of scientific publications in Peru, these three universities contribute 14%, 12%, and 3%, respectively.

## 4. Discussion

There is no doubt that the current University Reform in Peru is fostering a productive era. Before the new University Law (2014), Peruvian universities mainly trained professionals, neglecting research. Indeed, before this law, some university professors had no scientific publications and much less carried out research of significance in the various professions, thereby plunging the country into total underdevelopment. Thus, similar to what occurred in Ecuador and other Latin American countries, restructuring of the university system has regulated public and private universities leading to substantial improvement in the educational quality that students currently receive [13, 14].

This scientific development, especially of dental schools, seems to have allowed the dental community to successfully enter national and international capitalist markets. All of this is due to the success of the Latin American university reform (Peru, Ecuador, Chile, and Colombia). The results obtained with this bibliometric study demonstrate the effectiveness of the country's educational policies with a large amount of resources having been invested through institutions such as SUNEDU and CONCYTEC, allowing the new University Law to provide sustainability in the frameworks of science and research and thereby favor the training and retaining of the best researchers in the country [13, 14]. These public institutions were created to protect the rights of young people to receive a quality university education and, in this way, improve their academic skills [13].

The results obtained demonstrate that the scientific production of both public and private Peruvian universities has notably increased since the promulgation of the new University Law in 2014. Nonetheless, within the limitations of this study, other possible uncontrolled confounding factors may have influenced this causal relationship. The highest scientific output by private universities was found with articles published from 2014 to 2019. This might be explained by the changes in the policies of the public and private universities, generating economic incentives for publication, hiring research professors with better research profiles as well as empowerment and implementation of multifunctional laboratories for the execution of new research projects, among other reasons.

There is little bibliometric literature with Peruvian bibliometric data on dentistry, and thus, this is a pioneering study that reports the scientific output of the country's dental schools. For example, in the studies by Vioque et al. and Pereyra-Elías et al. evaluating the production of medical theses in indexed international journals, it was found medical students/residents mainly published their results in local magazines, likely because they were not aware of different sites for publication of their articles [15, 16]. In the present study, the theses publications were predominantly from the specialty of internal medicine and infectious-contagious diseases. This is mainly due to the UPCH being characterized worldwide by research on topics related to infectious and tropical diseases with high-quality research and training programs [17].

According to Yüksel et al. [18], thesis development in Turkey is important, above all for students to complement their medical experience. For this reason, they investigated the publication rates by university students between 2008 and 2011 and reported that 114 of 229 dissertations (49.7%) had been published. Then, they concluded that although the dissertation publication rate was acceptable, it could be improved. Similarly, according to Mayir et al. [19], knowledge cannot be diffused if it is not published in a scientific journal. However, the publication rate of thesis dissertations in journals is not high, and they concluded that scientific publications were insufficient, with only 22% in the Science Citation Index journals (WoS) and, consequently, the citation rate was also low, despite an increase in publications in internationally indexed journals and significant changes in publication dynamics. [20].

According to the study by Herrera-Añazco et al. [21], it is important to determine the scholarly output of the vice-chancellors for research in Peruvian medical schools. They, therefore, made searches in all the Peruvian universities with medical schools including 28 vice-chancellors of research. The average frequency of publication by the research vice-chancellors was 1.71, with a number of citations of 23.1. These results are somewhat similar to those of the present study in which the average number of articles published by public and private schools of odontology from 2015 to 2020 was similar, with the highest scientific production during that period of time being by private universities. Nonetheless, Herrera-Añazco et al. concluded that the scholarly output of the vice-chancellors for research was still insufficient, thereby questioning their leadership capacity in research.

This can likely be explained according to what was described by Mayta-Tristán et al. [22] who reported that promulgation of the novel University Law 30220 in 2014 led to compulsory institutional licensing of all Peruvian colleges, with medicine being one of the first programs to receive licensing. Bibliometrics serves as a tool to quantify scholarly output in indexed databases, the impact H index, and results of the national medical tests. However, in Peruvian dental schools, these same indicators are not managed. Similarly, it is important to note that the scholarly output of Peruvian medicine reported in the databases is also very poor, albeit notably increasing, especially in Lima [23]. On the other hand, it is essential to recognize that incentives for publication can increase the scientific production of institutions. Support programs may be used as a mechanism for transferring small amounts of payments to authors, although considerable improvements in the impact and quality of the manuscripts published should also be considered [24–27]. According to Nieto-Gutierrez et al. [24] in Peru, there are only 6 universities with medical schools that offer incentives for scientific publication, thereby indicating that it is necessary to implement this type of program in other schools, especially public institutions, to encourage an increase in publications while maintaining surveillance against possible faults to the scientific community.

The main limitation of this research was that only the scientific production of the Peruvian dental schools in Scopus was evaluated, and some dental schools may have publications in other databases that were not reviewed. Another great limitation of this bibliometric study was that the scholarly output of both universities that are and are not licensed by SUNEDU should have been evaluated in order to expand the sample of faculties in Peru. Thus, the main limitations of this type of bibliometric study are similar to those previously described by several authors [22–24] who described the main limitations of health science students as an absence of work value, poor awareness as to where to publish, lack of publication culture, and lack of support from mentors, among other aspects.

Nonetheless, the main strengths of this bibliometric study were that, as a pioneer in this area, the scientific production of dental faculties of private universities versus state universities during a recent period is accurate in Scopus because it is one of the most prestigious, complete, and recognized bases in medical science [28]. For this reason, the results described allow the corresponding authorities to improve, enhance, and sensitize the Peruvian dental community to continue improving quality scientific production in conjunction with the basic conditions of educational quality in the Peruvian university system. Finally, our results allow recommending that the results be complemented with research that can be analyzed in other indexed databases such as Web of Sciences (WOS), Medline, EMBASE, and Scielo, in order to determine the production and total impact of publications in dentistry in Peru and Latin America. Finally, complementary analyses are needed to determine if the work of SUNEDU has really influenced the increase in scientific production as a consequence of the assurance of basic conditions of educational quality.

## 5. Conclusions

Within the limitations and scope of the results of this study, it is concluded that there was a notable increase in scientific production in public and private dental schools. During the period of 2014–2019 after the promulgation of the new University Law 30220 in Peru, UPCH, UNMSM, and UCSUR are the top 10 institutions leading the ranking. Some factors such as economic incentives for publication, improvements of infrastructure, and the hiring of teachers with a research profile could boost scientific production in Peru and thereby ensure the quality of education and enhance national scientific production.

## Figures and Tables

**Table 1:** Scholarly output in public and private Faculties of Dentistry by the amount of international, national, and institutional collaboration (2014–2019).

Metric	%	Scholarly	Citations	Citations per publication	Field-weighted citation impact
International collaboration	54.9	185	4949	26.8	5.4
Only national collaboration	17.8	60	43	0.7	0.3
Only institutional collaboration	19.6	66	68	1	0.4
Single authorship (no collaboration)	7.7	26	28	1.1	0.3

Source: Scopus/SciVal database.

**Table 2:** Scholarly output by Scopus Source (2014–2019) of all public and private Peruvian Faculties of Dentistry.

Scopus source	Scholarly output	Citations	Authors	Citations per publication
Journal of Oral Research	41	13	94	0.3
American Journal of Orthodontics and Dentofacial Orthopedics	21	24	32	1.1
Revista Española de Cirugía Oral y Maxilofacial	11	6	16	0.0
Revista Cubana de Estomatologia	10	0	33	0.1
International Journal of Dentistry	9	6	42	1.6
Educacion Medica	8	19	11	0.9
Journal of Contemporary Dental Practice	7	2	30	0.7
Revista Peruana de Medicina de Experimental y Salud Pública	7	6	22	0.5
Dental Press Journal of Orthodontics	7	15	13	0.8
Brazilian Dental Journal	6	23	34	1.3

Source: Scopus/SciVal database.

**Table 3:** Share of publications per journal quartile by CiteScore Percentile of all public and private Peruvian Faculties of Dentistry (2014–2019).

CiteScore quartile	2014	2015	2016	2017	2018	2019	Overall
Q1 (top 25%)	11	11	13	18	18	20	91
Q2 (top 26%–50%)	6	6	10	14	26	33	95
Q3 (top 51%–75%)	1	6	3	8	4	14	36
Q4 (top 76%–100%)	8	5	11	14	23	43	104
Without Q	2	1	2	2	2	2	11
Total	28	29	39	56	73	112	337
%	2%	1%	2%	2%	2%	3%	2%
Peru	17 34	2080	2466	2966	3504	4366	17116
Dentistry Schools	718	994	1150	1226	1468	1810	7316
%	4%	3%	3%	5%	5%	6%	5%

Source: Scopus/SciVal database.

**Table 4:** Top 10 in scholarly output in Scopus of all the Peruvian public and private dental schools (2014–2019).

Institution	Scholarly output	Citations	Authors	Citations per publication
Universidad Peruana Cayetano Heredia	79	410	85	5.2
Universidad Nacional Mayor de San Marcos	53	137	79	2.6
Universidad Científica del Sur	49	129	52	2.6
Universidad de San Martín de Porres	40	118	51	3
Universidad Peruana de Ciencias Aplicadas	21	50	40	2.4
Universidad Privada Antenor Orrego	18	16	11	0.9
Universidad Nacional de Trujillo	16	7	18	0.4
Universidad Privada San Juan Bautista	8	5	25	0.6
Universidad Nacional Federico Villarreal	6	4	7	0.7

Source: Scopus/SciVal database.

**Table 5:** Top 3 authors of scholarly output in Scopus of all Peruvian public and private dental schools (2014–2019).

Author	Scholarly output	Most recent publication	University	Citations per publication	h-index	International collaboration
Arriola-Guillén, Luis	20	2019	UCSUR	1.8	5	17
Mayta-Tovalino, Frank	14	2019	UNMSM UCSUR	0.4	3	1
Mendoza-Azpur, Gerardo	14	2019	UCSUR	2.4	4	13

Source: Scopus/SciVal database.

**Table 6:** Scholarly output and international collaboration (%) according to publications in top 25% Journal Percentiles by CiteScore Percentile (%) = Q1 output in top 25% Citation Percentiles (%) (2014–2019).

Institution ID	Institution	Scholarly output	International collaboration (%)	Publications in top 25% Journal Percentiles by CiteScore Percentile (%)	Output in top 25% Citation Percentiles (%)
605001	Universidad Peruana Cayetano Heredia	79	78.5	51.9	36.7
605002	Universidad Nacional Mayor de San Marcos	53	39.6	15.4	18.9
701187	Universidad Científica del Sur	49	79.6	26.5	20.4
701190	Universidad de San Martín de Porres	39	35.9	16.2	17.9
701181	Universidad Peruana de Ciencias Aplicadas	21	38.1	22.2	14.3
714115	Universidad Privada Antenor Orrego	18	27.8	0	0
701197	Universidad Nacional de Trujillo	16	6.2	0	12.5
715762	Universidad Privada San Juan Bautista	8	12.5	0	25
706992	Universidad César Vallejo	6	0	0	0
701199	Universidad Nacional Federico Villarreal	6	16.7	16.7	16.7
708651	Universidad Católica de Santa María	5	40	20	20
717674	Universidad Nacional Hermilio Valdizán	3	0	0	0
717661	Universidad Católica Santo Toribio de Mogrovejo	2	0	0	0
701196	Universidad Nacional de la Amazonía Peruana	1	100	0	0
708650	Universidad Nacional Jorge Basadre Grohmann	1	0	0	0

Source: Scopus/Scival database.

## Data Availability

The data used in the statistical analysis of this study will be available upon authorization of the corresponding author.

## Supplementary Materials

Supplementary Materials Appendix S1: list of subterms. Appendix S2: search formula used. Appendix S3: list of institutions with their AF-IDs. Appendix S4: scientific production per year of each university (2014–2019) and its impact with respect to the total scientific production in Peru. .
