# Supplementary material for: Bibliometric Study of the National Scientific Production of All Peruvian Schools of Dentistry in Scopus
Source: Int J Dent. 2021 Apr 16;2021:5510209. doi: 10.1155/2021/5510209 (PMC8068529; doi:10.1155/2021/5510209)
Supplement: Supplementary Materials — Appendix S1: list of subterms. Appendix S2: search formula used. Appendix S3: list of institutions with their AF-IDs. Appendix S4: scientific production per year of each university (2014–2019) and its impact with respect to the total scientific production in Peru. . [file 5510209.f1.docx]

**Supplementary material**

Additional information supporting the results of this bibliometric study can be accessed at the end of this article.

**Appendix S1: List of Subterms**

| Dentistry(all) | 3500 |
| --- | --- |
| Dentistry (miscellaneous) | 3501 |
| Dental Assisting | 3502 |
| Dental Hygiene | 3503 |
| Oral Surgery | 3504 |
| Orthodontics | 3505 |
| Periodontics | 3506 |

**Appendix S2: Search formula used**

( ( ( SUBJTERMS ( 3500 OR 3501 OR 3502 OR 3503 OR 3504 OR 3505 OR 3506 ) ) OR ( TITLE-ABS-KEY ( odontolog* OR dentist* OR endodontic* OR orthodontic* OR periodontic* OR prosthodontic* OR dental OR odontometr* OR “dental bonding*” OR “anesthesia dental” OR “dental equipment*” OR “Oral hygiene index*” OR “dental occlusion*” OR “dentistry operative*” OR “diagnosis oral*” OR “esthetics dental*” OR “oral medicine*” OR “oral surgery*” OR “mouth rehabilitation*” OR odontometr* OR “pathology oral*” OR “preventive dentistr*” OR “dental technolog*” OR “tooth preparation*” OR “evidence-based dentistr*” OR “forensic dentistr*” OR “geriatric dentistr*” OR “occupational dentistr*” OR “school dentistr*” OR “public health dentistr*” OR “dental research” ) ) OR ( TITLE-ABS-KEY ( oral W/3 medicine ) OR TITLE-ABS-KEY ( oral W/3 surg* ) OR TITLE-ABS-KEY ( tooth W/3 preparation ) OR TITLE-ABS-KEY ( tooth W/3 remineralization ) ) ) ) AND ( PUBYEAR > 2013 ) AND ( EXCLUDE ( PUBYEAR , 2020) )

**Appendix S3: List of institutions with their AF-IDs**

| Institution ID | Institution | Sector | Country |
| --- | --- | --- | --- |
| 714116 | Universidad Continental | academic | Peru |
| 701175 | Instituto Nacional de Salud del Niño | government | Peru |
| 701176 | Instituto Nacional de Salud del Perú | government | Peru |
| 715867 | Seguro Social de Salud del Perú | government | Peru |
| 717674 | Universidad Nacional Hermilio Valdizán | academic | Peru |
| 701163 | Hospital Nacional Cayetano Heredia | medical | Peru |
| 701169 | Instituto de Investigación Nutricional | academic | Peru |
| 701174 | Instituto Nacional de Enfermedades Neoplásicas | government | Peru |
| 701195 | Universidad Nacional de Ingeniería | academic | Peru |
| 701202 | Naval Medical Research Unit Six | academic | Peru |
| 701400 | Universidad San Ignacio de Loyola | academic | Peru |
| 714110 | Universidad de Piura | academic | Peru |
| 717657 | Universidad Nacional Santiago Antúnez de Mayolo | academic | Peru |
| 717661 | Universidad Católica Santo Toribio de Mogrovejo | academic | Peru |
| 717663 | Hospital Regional Lambayeque | medical | Peru |
| 701165 | Hospital Nacional Edgardo Rebagliati Martins | medical | Peru |
| 701173 | Instituto Nacional de Ciencias Neurológicas | government | Peru |
| 701196 | Universidad Nacional de la Amazonía Peruana | academic | Peru |
| 701198 | Universidad Nacional del Altiplano de Puno | academic | Peru |
| 708650 | Universidad Nacional Jorge Basadre Grohmann | academic | Peru |
| 715066 | Consejo Nacional de Ciencia, Tecnología e Innovación Tecnológica | government | Peru |
| 715073 | Marina de Guerra del Perú | government | Peru |
| 715109 | Universidad Nacional Pedro Ruiz Gallo | academic | Peru |
| 716916 | Universidad Nacional del Centro del Perú | academic | Peru |
| 717665 | Asociación Civil Selva Amazónica | other | Peru |
| 717673 | Hospital Nacional Guillermo Almenara Irigoyen | medical | Peru |
| 718050 | Ministerio de Cultura, Perú | government | Peru |
|  |  |  |  |
|  |  |  |  |

**Appendix S4: Scientific production per year of each university (2014-2019) and its impact with respect to the total scientific production in Peru.**

| UUniversity /Country | **2014** | | **2015** | | **2016** | | **2017** | | **2018** | | **2019** | | **Overall** | |
| --- | --- | --- | --- | --- | --- | --- | --- | --- | --- | --- | --- | --- | --- | --- |
| Peru | 1735 | % | 2080 | % | 2467 | % | 2966 | % | 3502 | % | 4332 | % | 17082 | 100% |
| Universidad Católica De Santa María | 6 | 1% | 12 | 1% | 18 | 1% | 23 | 2% | 23 | 1% | 53 | 3% | 135 | 0% |
| Universidad Católica Santo Toribio de Mogrovejo | 3 | 0% | 4 | 0% | 8 | 1% | 14 | 1% | 6 | 0% | 6 | 0% | 41 | 1% |
| Universidad Científica del Sur | 20 | 3% | 76 | 7% | 130 | 10% | 66 | 5% | 97 | 6% | 134 | 7% | 523 | 0% |
| Universidad César Vallejo | 3 | 0% | 4 | 0% | 6 | 0% | 15 | 1% | 23 | 1% | 38 | 2% | 89 | 0% |
| Universidad de San Martín de Porres | 41 | 5% | 50 | 5% | 100 | 8% | 106 | 8% | 135 | 8% | 129 | 6% | 561 | 3% |
| Universidad Nacional de la Amazonía Peruana | 12 | 2% | 15 | 1% | 23 | 2% | 20 | 1% | 31 | 2% | 36 | 2% | 137 | 1% |
| Universidad Nacional de Trujillo | 13 | 2% | 16 | 2% | 43 | 3% | 46 | 3% | 63 | 4% | 79 | 4% | 260 | 3% |
| Universidad Nacional del Altiplano de Puno | 1 | 0% | 9 | 1% | 12 | 1% | 20 | 1% | 21 | 1% | 43 | 2% | 106 | 1% |
| Universidad Nacional Federico Villarreal | 8 | 1% | 13 | 1% | 24 | 2% | 32 | 2% | 38 | 2% | 35 | 2% | 150 | 1% |
| Universidad Nacional Hermilio Valdizán | 1 | 0% | 2 | 0% | 3 | 0% | 5 | 0% | 8 | 0% | 22 | 1% | 41 | 2% |
| Universidad Nacional Jorge Basadre Grohmann | 7 | 1% | 3 | 0% | 3 | 0% | 1 | 0% | 9 | 1% | 19 | 1% | 42 | 1% |
| Universidad Nacional Mayor de San Marcos | 238 | 31% | 287 | 28% | 295 | 23% | 377 | 27% | 441 | 27% | 458 | 23% | 2096 | 1% |
| Universidad Peruana Cayetano Heredia | 324 | 43% | 372 | 36% | 405 | 31% | 420 | 30% | 424 | 26% | 490 | 24% | 2435 | 0% |
| Universidad Peruana de Ciencias Aplicadas | 71 | 9% | 143 | 14% | 150 | 12% | 141 | 10% | 232 | 14% | 383 | 19% | 1120 | 0% |
| Universidad Privada Antenor Orrego | 4 | 1% | 14 | 1% | 34 | 3% | 45 | 3% | 33 | 2% | 28 | 1% | 158 | 12% |
| Universidad Privada San Juan Bautista | 2 | 0% | 0 | 0% | 8 | 1% | 8 | 1% | 11 | 1% | 21 | 1% | 50 | 14% |
| Total | 760 | 100% | 1028 | 100% | 1299 | 100% | 1385 | 100% | 1630 | 100% | 2029 | 100% |  | 7% |
|  |  |  |  |  |  |  |  |  |  |  |  |  |  | 1% |
|  |  |  |  |  |  |  |  |  |  |  |  |  |  |  |
